# Over‐ and under‐treatment of TB patients in Eastern China: an analysis based on health insurance claims data

**DOI:** 10.1111/tmi.13287

**Published:** 2019-07-29

**Authors:** Wenhui Mao, Weixi Jiang, Carol Hamilton, Hui Zhang, Fei Huang, Henry Lucas, Shitong Huan, Shenglan Tang

**Affiliations:** ^1^ Duke Global Health Institute Duke University Durham NC USA; ^2^ Global Health Research Center Duke Kunshan University Kunshan China; ^3^ Duke University School of Medicine Durham NC USA; ^4^ National Center for TB Control and Prevention, China CDC Beijing China; ^5^ Institute of Development Studies Sussex University Brighton UK; ^6^ Bill & Melinda Gates Foundation Beijing China

**Keywords:** tuberculosis (TB), over‐treatment, under‐treatment, health insurance, financial burden, tuberculose, sur‐traitement, sous‐traitement, assurance santé, charge financière

## Abstract

**Objective:**

Poor compliance with existing guidelines for tuberculosis (TB) care and treatment is an issue of concern in China. We assessed health service use by TB patients over the entire treatment process and compared it to the recommended guidelines.

**Methods:**

We collected insurance claims data in three counties of one province of Eastern China. Patient records with a diagnosis of ‘pulmonary TB’ in 2015 and 2016 were extracted. Treatment duration, number of outpatient (OP) visits and hospital admissions, as well as total cost, out‐of‐pocket (OOP) payments and effective reimbursement rates were analysed.

**Results:**

A total of 1394 patients were included in the analysis. More than 48% received over the 8 months of treatment that TB guidelines recommend, and over 28% received less. 49% of Urban and Rural Resident Basic Medical Insurance (URRBMI) TB patients were hospitalised while 30% of those with Urban Employee Basic Medical Insurance (UEBMI) had at least one admission. Median total cost for patients with hospital admission was almost 10 times that of patients without. By comparison, the average OOP was 5 times higher. UEBMI patients had a shorter treatment period, more outpatient visits but considerably fewer hospital admissions than URRBMI patients.

**Conclusions:**

We found an alarming extent of TB over‐ and under‐treatment in our study population. There is an urgent need to improve compliance with treatment guidelines in China and to better understand the drivers of divergence. Extending the coverage of health insurance schemes and increasing reimbursement rates for TB outpatient services would seem to be key factors in reducing both the overall cost and financial burden on patients.

## Abstract (fr)

**Objectif:**

Le mauvais respect des directives existantes en matière de soins et de traitement de la tuberculose (TB) est un sujet préoccupant en Chine. Nous avons évalué l'utilisation des services de santé par les patients TB tout au long du processus de traitement et l'avons comparée aux directives recommandées.

**Méthodes:**

Nous avons collecté des données sur les réclamations d'assurance dans trois comtés d'une province de l'est de la Chine. Les dossiers de patients avec un diagnostic de «TB pulmonaire» en 2015 et 2016 ont été extraits. La durée du traitement, le nombre de visites ambulatoires et d'hospitalisations, ainsi que le coût total, les paiements directs et les taux de remboursement effectifs ont été analysés.

**Résultats:**

1.394 patients ont été inclus dans l'analyse. Plus de 48% ont reçu plus de 8 mois du traitement recommandé par les directives TB et plus de 28% en ont reçu moins. 49% des patients TB résidents urbains et ruraux de l'assurance médicale de base (URRBMI) ont été hospitalisés, tandis que 30% de ceux avec une assurance médicale de base des employés urbains (UEBMI) ont eu au moins une admission. Le coût total moyen pour les patients hospitalisés était près de 10 fois plus élevé que celui des patients non hospitalisés. En comparaison, le payement direct moyen était 5 fois plus élevé. Les patients UEBMI ont eu une période de traitement plus courte, plus de visites ambulatoires mais beaucoup moins d'hospitalisations que les patients URRBMI.

**Conclusions:**

Nous avons trouvé une étendue alarmante de sur‐ et sous‐traitement de la TB dans notre population d’étude. Il est urgent d'améliorer le respect des directives de traitement en Chine et de mieux comprendre les facteurs de divergence. L'extension de la couverture des schémas d'assurance santé et l'augmentation des taux de remboursement des services ambulatoires pour la TB sembleraient être des facteurs essentiels pour réduire à la fois le coût global et la charge financière pour les patients.

## Introduction

The estimated incidence rate of tuberculosis (TB) in China in 2016 was 64 per 100 000 population 1, and recent reports have noted a marked regional disparity: TB prevalence appears to be 1.6 times higher in rural areas 2. Significant government subsidies are provided to TB‐designated hospitals to subsidise the provision of free essential TB care, including first‐line anti‐TB medicines, smear tests and chest X‐rays. Recently, extended coverage of the Basic Medical Insurance Schemes1The Basic Medical Insurance Schemes include the Urban Employees’ Basic Medical Insurance (UEBMI), Urban Residents’ Basic Medical Insurance (URBMI) and New Corporative Medical Scheme (NCMS). Recently, the URBMI and the NCMS have been integrated into Urban and Rural Residents’ Basic Medical Insurance (URRBMI) in most provinces of China., as part of China's ongoing health system reform towards universal health coverage, has also improved access to quality care and reduced the overall cost of TB treatment and financial burden of TB patients 2, 3, 4, 5.

Despite the increasing investment in TB care, poor compliance with existing guidelines for TB care and treatment is an issue of concern 6, 7, 8 that might lead to a higher incidence of drug‐resistant TB 9, 10. Lack of adherence to treatment standards results in both over‐ and under‐provision of TB services in Chinese hospitals 11. For example, most TB cases can safely be treated without hospitalisation, yet the reported hospital admission rate for TB patients ranges from 39% to 83% by different studies 12, 13, 14. This may be explained by the fact that inpatient services have considerably better insurance coverage than outpatient (OP), with the extent varying between local schemes, and there is thus an overall incentive for TB patients to use inpatient services 2, 15, 16, 17.

Evidence about TB patients' use of services mostly comes from reviews of clinical records or patient surveys 2, 3, 12, 14, 17. The first option is problematic in that it requires the collaboration of all facilities where TB patients may be treated. While this should be confined to ‘designated TB hospitals’, patient survey data indicates that this certainly cannot be assumed 18. Incomplete data are often an issue, particularly regarding OP visits, and TB prevalence is highest in poor rural areas where hospital information systems tend to be least reliable. Patient surveys raise serious issues in terms of data reliability, particularly for financial information, and it is often difficult to define the appropriate sampling population, given that patients are not restricted to seeking treatment in their county of residence. In both cases, data on overall service utilisation (inpatient and outpatient) over the extended period of TB treatment 5, 19 tend to be unreliable.

We aimed to address this limitation by analysing insurance data, where time‐series information is available on all those seeking reimbursement and where there is a reasonable expectation that financial data will be reliably recorded. We first present the demographic characters of the study population. Then, we compare the actual situation in terms of treatment duration, number of OP visits and hospital admissions among TB patients to those recommended by national treatment guidelines. Finally, we describe the financial burden on patients with different patterns of service use and covered by the two main insurance schemes.

## Methods

### Study site and insurance details

We selected three counties from a prefecture (we will use ‘Q’ for this prefecture, and ‘A’ ‘B’ and ‘C’ for the three counties for reasons of confidentiality) in one province in Eastern China where the prefecture‐level insurance management agency has claim records for all enrollees and allowed us to comprehensively analyse the use of services by TB patients. In 2017, the prefecture had 2.2 million residents and the average GDP per capita was 63 492 CNY (9,406USD) 20. In all three counties, patients can seek services at county‐ or prefecture‐level TB‐designated hospitals.

The three counties implemented the same health insurance schemes in 2015 and 2016. The URRBMI covers unemployed residents (including students) and provides 65% reimbursement for hospital admissions in county hospitals and 60% in prefecture hospitals after an 800 CNY (124USD) deductible. It provides only 7 CNY reimbursement for each OP visit in county‐level hospitals, while the reported average cost of outpatient treatment is 800–2000 CNY (124‐310 USD 13). The UEBMI enrols employees from the formal sector and large private enterprises and provides a more generous benefit package than the URRBMI. For hospital admissions, the reimbursement rate is 84% after the deductible payment of 800 CNY, while OP treatment has a reimbursement rate of 50% for county‐level hospitals with a 300 CNY deductible. Enrollees can claim at most 3000 CNY for outpatient service expenditure per year 21, 22, 23, 24 (More details of the insurance schemes can be found in Appendix 1).

### Working definition for over‐ and under‐treatment

Since 2009, the WHO has recommended that new patients with pulmonary TB should be treated with a 6‐month regimen containing rifampicin (2 months of isoniazid, rifampicin, pyrazinamide and ethambutol followed by 4 months of isoniazid and rifampicin, 2HRZE/4HR). Previously treated patients should receive treatment for 8 months of first‐line drugs (2 months of isoniazid, rifampicin, pyrazinamide, ethambutol and streptomycin, followed by 1 month of isoniazid, rifampin, pyrazinamide and ethambutol, followed by 5 months of isoniazid, rifampin and ethambutol, 2HRZES/1HRZE/5HRE). The Chinese National TB guidelines also recommend monthly OP visits for TB patients while they are receiving treatment 10. Accordingly, we define under‐treatment as patients who had fewer than 6 OP visits and over‐treatment as patients who had more than 8 visits.

### Data collection

We collected data from the health insurance claim system managed by the Department of Human Resources and Social Security (HRSS) of Prefecture Q. The database covers all services used by enrollees. All records of OP visits and hospital admissions from January 1, 2015 to December 31, 2016 with primary diagnosis (discharge diagnosis for hospital admissions) of ‘pulmonary tuberculosis’ were extracted.

### Eligibility

Our analysis covered those patients whose first insurance claim was made before June 1, 2016 or whose last claim was made after May 31, 2015 to allow time for treatment completion. Given that the database included TB suspects, those patients who only had 1 or 2 records and less than 1 month's treatment were regarded as TB suspects and therefore excluded. Patients with a diagnosis of MDRTB, drug‐resistant TB or relapse TB were also excluded.

Using the above criteria, 3538 patients with a TB diagnosis were identified. In total, 1317 were excluded because either their first record was later than June 1, 2016 or last record was before May 31, 2015. A total of 718 patients with only 1 or 2 records and less than 1 month's treatment were excluded. A total of 109 more patients were removed for having records with a DR, MDRTB or relapse TB diagnosis. Finally, 1394 patients were included in our analysis. The percentage of TB cases with MDR/RR TB was 7.25% in our study population, which is very close to WHO estimates (7.1%) 25.

### Measurements

Patient‐level data collected included age, gender, registered county, insurance type, name of hospital, date of visit, type of service (OP visit or hospital admission), total cost, effective reimbursement by insurance and OOP payment for each service use.

### Analysis

A unique patient ID was used to match and accumulate all the records of OP visits and hospital admissions for each individual. The interval between earliest and latest use of health service (including OP visits and hospital admission) was calculated to get treatment duration. Number of OP visits and hospital admissions were accumulated for each patient, as well as total cost, out‐of‐pocket (OOP) payment and reimbursement from insurance. We divided accumulated reimbursement from insurance by accumulated total expenditure to calculate the effective reimbursement rate for each patient. Stratified analysis was performed by type of insurance (URRBMI/UEBMI) and patient registered county, which may influence geographic and economic access to services. Statistical tests were conducted to identify significant differences (α = 0.05). Specifically, t‐tests were used for two‐group comparisons on continuous variables, for example age and total expense, one‐way ANOVA for continuous variables across three groups, and chi‐squared tests for categorical variables, such as number of OP visits.

We reported the means and selected distribution statistics for treatment duration, number of OP visits and hospital admissions. Considering the skewed distribution of financial indicators 26, we used medians for total cost, OOP and reimbursement rate. To observe the relationship between service use and financial burden on patients with different treatment durations, we also compared the number of OP visits, number of hospital admissions, total cost, OOP and reimbursement rate between patients with 6–8 months treatment and patients with more than 8 months treatment. We also compared the total cost, OOP and reimbursement rate between patients with and without hospital admissions to observe the influence of admission on the financial burden.

### Ethical considerations

This project is part of a larger project entitled ‘Monitoring, Learning and Evaluation for the Implementation of Comprehensive Models of TB Care and Control in China’. The protocol of study design and data collection tools was reviewed and approved by the Institutional Review Board of Duke University (IRB Approval code: 2017‐0768). In brief, the data extraction was performed by the Department of HRSS of Prefecture Q, the legally authorised government agency to collect, access and manage insurance claims data. Two authorised investigators have access to the data. Patient insurance registration number was the only identifiable information we collected to match service records at patient level and this number was deleted after re‐coding with a random number. No other identifiable data such as name or address of patients were collected. All results were presented at county or group level. Results of this analysis will be merged with other findings of the larger project and shared with national and local TB Programme Management Offices in China.

## Results

### Demographic characters of study population

We collected 1145 TB patients enrolled in URRBMI and 249 in UEBMI. As displayed in Table 1, over 65% were male, with no statistical difference between the two insurance groups. The average age of URRBMI was 60 years and of UEBMI patients 55 years (*P *< 0.05). Sex and age variables for patients with the same insurance differed moderately across counties but this was not statistically significant. (Table 1).

**Table 1:** Demographic characters of study population

	URRBMI			UEBMI		
	Number of patients	% of male	Age (mean)	Number of patients	% of male	Age (mean)
Total	1145	69.6%	59.71	249	65.1%	54.63
County A	444	73.4%	59.99	85	69.4%	53.54
County B	454	68.3%	59.56	94	60.6%	53.54
County C	247	64.8%	59.48	70	65.7%	57.41

Statistical test on % of male: *P*> 0.05 between URRBMI and UEBMI; *P*> 0.05 among three counties (URRBMI); *P*> 0.05 among three counties (UEBMI); Statistical test on Age: *P* < 0.05 between URRBMI and UEBMI; *P*> 0.05 among three counties (URRBMI); *P*> 0.05 among three counties (UEBMI).

### Use of services

The overall pattern of service use by patients with diagnosed drug‐susceptible TB in the three counties was characterised by long treatment duration, large variation in number of OP visits and high proportion of hospital admissions (Tables 2, 3, 4).

**Table 2:** Treatment duration (Months)

	URRBMI						UEBMI					
	Mean	Distribution of patients with different treatment duration, %					Mean	Distribution of patients with different treatment duration, %				
		<6 months (underuse)	6–8 months (recommended)	(8–12]	(12–18]	Over 18 months		<6 months (underuse)	6–8 months (recommended)	(8, 12]	(12–18]	Over 18 months
Total	8.70	31.2	20.5	25.0	16.5	6.8	8.94	28.1	22.9	24.9	16.5	7.6
County A	8.10	28.4	25.9	31.1	11.7	2.9	8.09	25.9	30.6	28.2	11.78	3.5
County B	10.29	23.6	18.1	22.5	23.4	12.6	10.66	22.3	18.1	20.2	23.4	16.0
County C	6.84	50.2	15.4	18.6	12.6	3.2	7.65	38.6	20.0	27.1	12.9	1.4

Statistical test on distribution of treatment duration: *P*> 0.05 between URRBMI and UEBMI; *P* < 0.05 among three counties (URRBMI); *P* < 0.05 among three counties (UEBMI).

**Table 3:** Outpatient visits

OP visits	URRBMI						UEBMI					
	Mean	Distribution of patients with different OP visits, %					Mean	Distribution of patients with different OP visits, %				
		3 or less (severe underuse)	4&5 (underuse)	6–8 (recommended)	9–12 (overuse)	13 or more (severe overuse)		3 or less (severe underuse)	4&5 (underuse)	6–8 (recommended)	9–12 (overuse)	13 or more (severe overuse)
Total	7.49	25.2	17.3	23.1	19.9	14.5	7.47	26.1	16.1	19.3	22.9	15.7
County A	7.47	13.8	22.6	31.5	23.5	8.6	7.73	12.9	24.7	24.7	21.2	16.5
County B	9.42	21.5	10.3	18.9	22.6	26.6	5.14	23.4	10.6	14.9	25.5	25.5
County C	4.00	53.9	19.8	14.6	8.1	3.6	7.47	45.7	12.9	18.6	21.4	1.4
Patients with less than 6 months' treatment												
Total	4.99	43.7	21.0	18.5	11.8	5.0	4.73	40.0	25.7	24.3	8.6	1.4
County A	6.10	31.0	23.0	19.8	18.3	7.9	5.05	22.7	50.0	18.2	4.6	4.6
County B	5.24	40.2	19.6	24.3	11.2	4.7	5.24	33.3	19.1	33.3	14.3	0
County C	3.64	59.7	20.2	12.1	5.7	2.4	4.73	59.3	11.1	22.2	7.4	0
Patients with 6–8 months' treatment												
Total	7.09	14.9	16.6	38.3	24.3	6.0	7.56	10.5	19.3	31.6	29.8	8.8
County A	6.59	10.4	17.4	51.3	20.9	0	7.46	7.7	23.1	34.6	23.1	11.5
County B	8.72	15.9	6.1	25.6	36.6	15.9	8.53	5.9	17.6	23.5	41.2	11.7
County C	5.05	26.3	36.8	26.3	7.9	2.6	6.57	21.4	14.3	35.7	28.6	0
Patients with more than 8 months' treatment												
Total	9.28	19.2	10.1	21.0	24.6	25.1	9.01	25.4	9.0	10.6	27.9	27.1
County A	8.82	7.4	12.8	32.5	32.0	15.3	9.51	10.8	10.8	21.6	29.7	27.0
County B	11.32	15.9	7.6	14.7	23.0	38.9	10.52	25.0	5.4	5.4	25.0	39.3
County C	4.05	57.7	11.8	12.9	11.8	5.9	5.45	44.8	13.8	6.9	31.0	3.5

Statistical test on distribution of OP visits: *P*> 0.05 between URRBMI and UEBMI: *P* < 0.05 among three counties (URRBMI); *P* < 0.05 between URRBMI patients with different treatment duration; *P* < 0.05 among three counties (UEBMI); *P* < 0.05 between UEBMI patients with different treatment duration.

**Table 4:** Hospital admission

Hospital admissions	URRBMI					UEBMI				
	Mean	Distribution of patients with different hospital admissions, %				Mean	Distribution of patients with different hospital admissions, %			
		0	1	2	3 or more		0	1	2	3 or more
Total	0.83	51.4	30.2	9.8	8.7	0.67	67.9	20.9	5.2	6.0
County A	0.77	44.1	40.1	11.0	4.7	0.68	56.5	35.3	3.5	4.7
County B	0.46	68.5	23.1	4.9	3.5	0.16	86.2	12.8	0	1.1
County C	1.62	32.8	25.5	16.6	25.1	1.34	57.1	14.3	14.3	14.3
Patients with less than 6 months' treatment										
Total	0.76	54.1	27.7	11.5	7.0	0.57	70.0	17.1	5.7	7.1
County A	0.58	57.1	29.4	11.9	1.6	0.50	64.6	27.3	4.6	4.6
County B	0.51	64.5	25.2	6.5	3.7	0.14	85.7	14.3	0	0
County C	1.15	41.9	28.2	14.5	15.3	0.96	63.0	11.1	11.1	14.8
Patients with 6–8 months' treatment										
Total	0.64	55.3	33.2	6.4	5.1	0.61	59.7	31.6	5.3	3.5
County A	0.68	46.1	43.5	7.8	2.6	0.62	42.3	53.9	3.9	0
County B	0.24	78.1	20.7	0	1.2	0.12	88.2	11.8	0	0
County C	1.39	34.2	29.0	15.8	21.1	1.21	57.1	14.3	14.3	14.3
Patients with more than 8 months' treatment										
Total	0.97	47.9	30.6	10.3	11.2	0.75	70.5	18.0	4.9	6.6
County A	0.95	35.0	44.8	12.3	7.9	0.84	62.2	27.0	2.7	8.1
County B	0.51	67.2	23.0	5.7	4.2	0.18	85.7	12.5	0	1.8
County C	2.42	18.8	20.0	20.0	41.2	1.76	51.7	17.2	17.2	13.8

Statistical test on distribution of hospital admission: *P* < 0.05 between URRBMI and UEBMI; *P* < 0.05 among three counties (URRBMI); *P* < 0.05 between URRBMI patients with different treatment duration; *P* < 0.05 among three counties (UEBMI); *P*> 0.05 between UEBMI patients with different treatment duration.

More specifically, the average treatment duration was over 8 months. We identified 28% patients receiving less than 6 months treatment while 49% were classified as over‐treatment cases. Among this over‐treatment group, one‐third completed treatment within one year, 22% continued for between 12 and 18 months, and 10% for over 18 months. The difference between patients with URRBMI and UEBMI was not significant, while patients from different counties had significantly different treatment durations (*P* < 0.05). County B's patients were treated on average for 10.3 months, County C's for 8.1 months and County A's for 6.8 months (Table 2).

The mean number of OP visits was 7.5 (Table 3). However, patients with 6–8 OP visits, as recommended, accounted for only 23% of URRBMI and 16% of UEBMI patients. 25% of TB patients had only 3 or fewer OP visits. Another 16% had 4 or 5, indicating that more than 40% of TB patients could be classified as under‐treated. However, 35% of patients were identified as over‐treated (Table 3).

TB patients in the studied counties also had high hospital admission rates. 49% of URRBMI TB patients were hospitalised during the study period. This proportion was significantly lower among UEBMI patients (*P* < 0.05), but 30% had at least one admission (Table 4). UEBMI patients tended to attend more OP visits than those covered by URRBMI (*P*> 0.05), but had less hospital admissions (*P* < 0.05). It is also noticeable that the number of OP visits was negatively associated with that of hospital admissions (*P* < 0.05). For example, County C had the highest average number of hospital admissions but the lowest average number of OP visits (Tables 2, 3, 4).

Surprisingly, when we analysed patients with different treatment durations, longer treatment periods did not necessarily lead to increased service use (Tables 3 & 4). A higher proportion of patients with longer treatment periods experienced both underuse and overuse of OP visits compared to those with shorter periods (*P* < 0.05). This may reflect the number of patients who were inconsistent with medication collection and ingestion, who required longer periods to undertake a given number of visits.

### Financial burden

Patients without hospital admission had lower average cost, OOP and reimbursement from insurance than those with hospital admission. Average cost for patients with a hospital admission was almost 10 times that for patients without, while OOP expenses were five times higher (Table 5).

**Table 5:** Total cost, out‐of‐pocket payment and reimbursement (median)

	URRBMI				UEBMI			
	# of patients	Total cost	OOP	Effective reimbursement rate (%)	# of patients	Total cost	OOP	Effective reimbursement rate (%)
Total	1145	3548	2610	16.4	249	2157	1355	39.8
Patients without hospital admission	580	960	903	3.7	169	1153	704	32.2
Patients with hospital admissions	557	10 109	4990	50.0	80	11 814	3973	64.1
Patients with less than 6 months' treatment								
Total	357	1940	1588	15.7	70	1482	683	40.8
Patients without hospital admission	193	641	639	3.4	49	1066	488	35.0
Patients with hospital admissions	164	9388	4330	54.4	21	14 597	3789	70.4
Patients with 6–8 months' treatment								
Total	235	2720	2290	8.2	57	2453	1649	46.5
Patients without hospital admission	130	1082	1046	3.9	34	1030	644	35.7
Patients with hospital admissions	105	8635	4238	49.1	23	9284	3684	64.4
Patients with over 8 months' treatment								
Total	553	4900	3124	25.8	122	2490	1589	36.6
Patients without hospital admission	265	1348	1264	3.8	86	1478	1081	29.4
Patients with hospital admissions	288	10 941	5985	48.3	36	12 287	4358	62.4

Statistical test on total expense: *P*> 0.05 between URRBMI and UEBMI; *P* < 0.05 among three counties (URRBMI); *P* < 0.05 between URRBMI patients with different treatment duration; *P* < 0.05 among three counties (UEBMI); *P*> 0.05 between UEBMI patients with different treatment duration.

Statistical test on OOP: *P* < 0.05 between URRBMI and UEBMI; *P* < 0.05 among three counties (URRBMI); *P* < 0.05 between URRBMI patients with different treatment duration; *P* < 0.05 among three counties (UEBMI); *P*> 0.05 between UEBMI patients with different treatment duration.

Statistical test on reimbursement rate: *P* < 0.05 between URRBMI and UEBMI; *P* < 0.05 among three counties (URRBMI); *P*> 0.05 between URRBMI patients with different treatment duration; *P* < 0.05 among three counties (UEBMI); *P* < 0.05 between UEBMI patients with different treatment duration.

The average exchange rate between 2015 and 2016: 1USD = 6.44.

Longer treatment was associated with higher average cost and OOP for URRBMI patients (*P* < 0.05) but the opposite was observed among UEBMI patients, though this was not statistically significant. Overall, UEBMI patients had lower OOP (*P* < 0.05) and higher reimbursement (*P* < 0.05) than URRBMI patients. The most significant gap between the two insurance schemes on reimbursement rate existed among patients without hospital admission. The reimbursement rates were not significantly different across the three counties, yet the average cost and OOP expenses did vary significantly (*P* < 0.05) (Table 5).

## Discussion

This study attempted to analyse a previously underutilised data source, insurance claims data maintained by agencies which now provide coverage to the great majority of the Chinese population. With the close collaboration of provincial and county officials, it was found that this source can be effectively used to address key health systems concerns that have proved difficult to research using traditional approaches.

The substantive findings demonstrate that over‐treatment of TB patients is common in the counties studied, as measured by three indicators. First, more than 49% received longer treatment than the 6–8 months recommended by the WHO. This finding is consistent with Yang and Xia's research showing that 40% of TB patients received extended treatment, while some 10% appeared to have been treated for over 3 years 27, 28. Second, half of TB patients had more OP visits than recommended. Third, nearly half of TB patients had at least one hospital admission. As argued by other studies, excessive hospitalisation is a common, serious and growing problem, with the hospitalisation rate for TB patients increasing by 185% between 1999 and 2009 29, 30. Inappropriate financial incentives for hospitals and ineffective supervision of compliance with national treatment guidelines are important factors contributing to this finding and deserve further investigation.

Concurrently, improved health insurance schemes may also have encouraged increased services use, especially in terms of hospital admissions, where reimbursement rates are much higher than for outpatient care. It is not surprising to see a clear demonstration of ‘moral hazard’ arising from the financial incentives given to both service users and providers 2, 17. It should be noted that another possible reason for apparent over‐treatment, persistent symptoms and/or microbiology, indicative of undiagnosed drug‐resistance, cannot be assessed here. The database does not support further investigation of this theory, but it should be further studied as a potentially very dangerous development.

About one‐third of patients were identified as under‐treated. High proportions of patients with more than 8 months treatment experienced both too few and too many OP visits compared to patients with 6–8 months. Some severe or complex cases may lead to longer treatment periods and/or more OP visits. Non‐compliance may be another factor. Those who do not take TB treatment drugs as instructed will typically attend outpatient clinics only when they have finished the medication supplied. As a result, non‐adherence could entail longer or shorter periods of treatment.

When comparing service use by TB patients with different insurance schemes, it is noticeable that URRBMI patients had significantly more hospital admissions than UEBMI patients. Li et al. 31 suggest that the relatively more generous coverage for hospital admission by insurance schemes incentivises patients to seek inpatient treatment for even minor health conditions. Our finding can be seen as an illustration of Li's theory. However, URRBMI patients were on average 4 years older than those covered by the UEBMI and might have more healthcare needs. Therefore, this observation needs to be interpreted with caution.

Demographic characteristics (age and gender) were homogeneous across the three counties. However, our study found that the patients from the three counties had a different service use patterns for TB treatment (i.e. OP visits *vs.* hospital admissions). Local health service regulations, supervisory and monitoring activities by local health authorities, and the preferences of health providers may be important factors contributing to this finding, which deserves further investigation.

Patients with longer treatment and hospital admission usually had higher average cost. For example, the median cost for patients with a hospital admission was almost 10 times that for patients without. Similar results can be found in Jia's work 9. To reduce the financial burden of TB treatment and the overall cost, it is essential to develop effective mechanisms ensuring the reduction of unnecessary hospital admissions. The current benefit coverage, particularly that offered by URRBMI, is not sufficient, especially for outpatient services. URRBMI patients, mainly the unemployed, elderly and students who are regarded as vulnerable, have to bear significantly higher OOP expenses for their treatment than UEBMI patients. This inequity calls for attention from both policymakers and the medical financial assistance programme (the safety net of the social security system in China).

This study has limitations. First, the records from insurance claims data did not have a diagnosis category to identify TB suspects, or any information on the period before confirmed diagnosis. As indicated, we attempted to allow for this by excluding patients with only 1–2 records and at most one month of treatment. This may underestimate the under‐treatment of TB patients if some of those excluded cases were TB patients who quit treatment soon after commencement. Second, deaths of patients during treatment are not recorded. However, given that the TB mortality rate in China was 2.6 per 100 000 population and TB case fatality ratio 0.04 in 2017^31^, such deaths would have had minimal impact on the proportion of patients under‐treated.

Third, services not covered by health insurance were not recorded in the database. However, the percentage of people in China not covered by any health insurance scheme is less than 5% 32. The overall use of services and resulting financial burden on TB patients are therefore underestimated. The dataset for this study lacked clinical indicators and has not been linked to the TB registration data. Further investigations involving clinical information such as the results of sputum smear tests may reveal other reasons for treatment disparities and are recommended. Finally, this study did not attempt to explore the reasons for non‐compliance. These have been considered at length in many of the studies identified in the introductory section, though further research may be required.

## Conclusions

Over‐ and under‐treatment are alarmingly common among our patient population. There is an urgent need to improve compliance with treatment guidelines in China and to better understand the drivers of divergence. Extending the coverage of health insurance schemes and increasing reimbursement rates for TB outpatient services would seem to be key factors to reduce both the overall cost and financial burden on patients.

## Declarations

The primary dataset generated and analysed during the current study is not publicly available due to individual privacy considerations. However, a modified dataset, using randomised individual identifiers to replace all identifiable information, is available from the corresponding author on reasonable request. The modified data set will be made open‐access after the project is finished (expected to be December 2019).

## Appendix 1

### Insurance policy in Prefecture Q, 2015 &2016.

**Table:**

	URRBMI	UEBMI
Financial contribution	Individual pay 230 CNY and government subside 460 CNY	Plan A:individual pay 1% and employer pay 5% of personal income (no personal medical saving account; Plan B: individual pay 2% and employer pay 8% of personal income
Benefit package		
Hospital admissions	Deductible: 400 CNY/800 CNY for grassroots/county and prefecture hospitals for first hospital admission within one year, 200 CNY/600 CNY for second and no deductible since then; Reimbursement rate: deductible‐max payment of insurance, 70%/65%/60% for grassroots/county/prefecture hospitals* Max payment of insurance: 150 000 CNY	Deductible: 800 CNY for first hospital admission within one year, 600 CNY for second and no deductible since then; Reimbursement rate: deductible‐60 000 CNY, 84%†; 60 000 CNY‐max payment of insurance, 87%†; Max payment of insurance: 210 000 CNY
Outpatient visits	Reimbursement rate: 40%/7CNY for grassroots/county hospitals* Max payment of insurance: 1500 CNY	Plan B's enrollees can use personal medical saving account; Deductible: 300 CNY Reimbursement rate: deductible‐max payment of insurance, 55%/50%* for grassroots/county hospitals; Max payment of insurance: 3000 CNY
Referral	Individual pay 5%/10%15% total expense first for services use in hospitals in other counties of Quzhou/hospitals in other prefectures of Zhejiang Province/hospitals in other provinces, then follow the same reimbursement policy described in “benefit package”	Individual pay 5%/15% total expense first for service use in hospitals in other prefectures of Zhejiang Province/hospitals in other provinces, then follow the same reimbursement policy described in “benefit package”

The average exchange rate between 2015 and 2016: 1USD = 6.44.

* 10% additional reimbursement for hospitals implementing Zero‐Mark up policy, 5% more for hospitals implementing National Essential Medicine Policy.

† 5% additional reimbursement for retirees.
